# Efficacy of immediate patient feedback in emergency psychiatry: a randomized controlled trial in a crisis intervention & brief therapy team

**DOI:** 10.1186/1471-244X-13-331

**Published:** 2013-12-05

**Authors:** Flip Jan van Oenen, Suzy Schipper, Rien Van, Robert Schoevers, Irene Visch, Jaap Peen, Jack Dekker

**Affiliations:** 1 Arkin, Klaprozenweg 111, 1033 Amsterdam, NN, The Netherlands; 2 Universitair Medisch Centrum Groningen, Hanzeplein 1, 9713 Groningen, GZ, The Netherlands; 3 Klinische Psychologie Vrije Universiteit van Amsterdam, de Boelelaan 1105, 1081 Amsterdam, HV, The Netherlands

**Keywords:** Patient feedback, Randomized controlled trial, Crisis intervention, Efficacy, Outcome monitoring

## Abstract

**Background:**

This study looks at the effect of immediate session-by-session feedback using short questionnaires for evaluating outcome of and alliance in the therapy. Research data strongly suggests that using this feedback informed treatment improves the outcome of therapy. However, until now, this method of Miller and Duncan has only been examined in clients (generally students) with mild problems and in partner counselling. The question addressed by this study is whether immediate feedback is also effective when applied during crisis intervention and subsequent brief therapy in a psychiatric patient population in emergency situations. It also looks at whether 'feedback-informed treatment' affects the quality of the alliance.

**Method/Design:**

To test the hypotheses, all patients seeking help from the Crisis Intervention & Brief Therapy Team over a two-year period will be followed throughout their treatment up to a maximum of six months and a follow-up period of three months after ending the treatment. Patients are randomly assigned to two conditions: treatment without feedback and treatment with immediate feedback for each session. The therapists all operate in both conditions and so they deliver both treatments. An estimated total of 180 patients, aged 18 years and over, will be included in the study.

**Discussion:**

The aim of this study is to make clear whether, and to what extent, systematic feedback from the patient in this target group during therapy determines the course and outcomes of therapy. We also look at whether, and to what extent, the quality of the alliance and the motivation of the person delivering treatment with respect to the instruments play a role.

**Trial registration:**

NTR3168

## Background

Principal research showed, in a range of meta-analyses, that all bona fide psychotherapy treatments are, in broad terms, equally effective [1-7]. This finding, combined with economic concerns, led to a search for methods to improve treatment outcomes in other ways than the traditional evidence-based research. Out of this search, a new paradigm for evaluating psychotherapy came forward, introduced by Howard [8]: *patient focused research*. Central in this type of research is monitoring, modelling and predicting individual treatment progress and providing feedback about this information to therapists (and patients) during the course of treatment [8-10]. In this 'feedback-informed treatment' [11], therapists are supposed to identify patients at risk of treatment failure in an early phase and to make changes to their approach when necessary [9,12-16].

### Patients at risk of failure

When patients benefit from therapy, this generally happens in an early phase of treatment [17,18]. Several authors have looked at the issue of whether 'early symptom change', as experienced by the patient, is a feasible predictor of a good outcome in given patient-therapist combinations [9,15,19,20]. Lambert [9] found that in 84% of early changers subsequent therapy was positive; approximately 25% of the clients actually underwent a dramatically rapid improvement that –usually- led to sustained recovery. Howard et al. [14] found not only that change occurred earlier rather than later in the course of treatment, but also that the absence of early change diminished the probability of symptom reduction and improved well-being at the end of treatment. Brown et al. [12] found that when there is no improvement after three sessions generally no improvement will be achieved over the entire course of treatment; they also found that patients who report deterioration by the third session have a drop-out risk twice as high as patients who report improvement. An improvement in well-being in the first 3–5 sessions therefore has predictive value for the course of therapy. Also more specific patterns of early change, slow and fast have been identified [21].

### Results of feedback

In the first studies, feedback about symptom change was only given to the therapist. In a meta-analysis of three studies Lambert et al. [22] concluded that adding feedback to a treatment led to better outcomes, reporting an average Effect Size (ES) of 0.39.

Later, different forms of feedback have been developed providing both therapist and client with information. A meta-analysis of five studies [9] also reported a positive effect: fewer patients deteriorated in the feedback condition – 20% as opposed to 12% – and that more patients improved (defined as achieving clinically significant change) - 22% versus 45%-.

Several studies have been performed to establish which form of feedback offers the best results. Most feedback research is focused on the early identification of patients who are 'not on track' or whose scores develop in ways that differ from the expected scores in a negative way [9,14,16,23].

A comparison of several forms of feedback was made by Knaup [24] in a meta-analysis, including a total of twelve studies. Here also, the effect - at least in the short-term- on health outcomes proved to be significant in all feedback studies. However, the average effect size was lower (0.10) than in the earlier meta-analyses, and the effect could not be demonstrated during follow-up measurement. An important finding was that feedback was more effective when information about (lack of) patient progress was supplied, feedback was given frequently (more than twice) and given to both patient and therapist. It can also be concluded from this meta-analysis that the main effect of feedback results from identifying the Not On Track group in time [25].

### Feedback instruments

The feedback studies that meet the requirements that can be derived from the findings of Knaup as mentioned above, are the studies using the Outcome Questionnaire 45 (OQ45) of Lambert and the Partners for Change Outcome Management System (PCOMS) of Miller and Duncan [26,27]).

In the most recent meta-analysis, Lambert & Shimokawa [28] performed a meta-analysis of the effects of both the OQ45 and PCOMS, including a total of 9 randomized controlled trials. This study comprises six randomized studies on the OQ45 and three randomized studies on PCOMS. The latter concerning individual therapy in a student counselling university setting [29], and couples therapy in the context of an (American) university therapy training programme [30], respectively a (Norwegian) family counselling agency [31].

Lambert & Shimokawa found effects sizes varying from .23 to .33. They concluded that the number of psychotherapy patients who deteriorate (5–10% in adult therapy, 14–24% in child psychotherapy, in routine care) can be cut in half by use of these systems. Here also, the main effect comes from identifying Not On Track clients in good time. However, in some of the studies analyzed also beneficial effects were reported for ‘On Track’ clients [31-33].

### Limitations of studies: differences in setting and instruments

However, a limitation of these studies is that they are performed in psychotherapeutic settings and in populations with only mild distress and not suffering from psychiatric disorders. De Jong recently performed two feedback studies with the OQ45 in a naturalistic setting including patients with various psychiatric disorders. In the first study [34], for the full sample no beneficial effect of feedback was found and there was no effect of feedback on patients being Not On Track (NOT) either. In NOT cases a positive significant effect was found only when therapists indicated that they actually used the feedback. Feedback in this study however was only given to the therapist and not to the client, and not on a session-by-session basis.

In the second study [35], immediate feedback to only therapists was compared to feedback to both therapist and patient (vis-à-vis a non-feedback condition). Though feedback was applied each session, no overall effect was found either. However, applying feedback appeared to be preventive of negative change for NOT cases in short-term therapies (d = 1.28 after 35 weeks) but this effect was not strong enough to bend the course of treatment in the direction of positive change. Feedback was most effective when applied to both client and therapist. The findings that feedback was most effective for clients who were Not On Track and when feedback is supplied to both client and therapist, match with the conclusions of Lambert [9] and Knaup [24]. However, the study of De Jong was performed in a population with only mild distress (mean base line OQ45 69.2 resp. 74,6), comparable to Lamberts studies (mean OQ45 68–78), excluding patients in crisis and patients with a psychotic disorder.

Another limitation of all the studies mentioned is that the applied feedback measure was identical to the outcome measure; there was no use of an additional outcome measure independent of the feedback instrument.

Taking into account this state of affairs, it is still an open question whether feedback instruments should be implemented in daily psychiatric practice. Further studies should be conducted in a psychiatric population focusing on clients in severe distress, and using outcome measures that are not identical to the feedback measures. Such a study is provided for in this design.

### The role of the alliance

Repeatedly, the literature has shown that the therapeutic alliance is an important factor in psychotherapy [36]. There is some evidence that various measures of early (relationship-related) interpersonal in-session processes account for at least 8% to 14% of explained outcome variance [36,37].

Patient ratings of the therapeutic relationship between Sessions 3 and 5 provide reasonable predictions of treatment outcome [38]and a low score for the quality of the alliance emerged as a predictor for premature termination of treatment [39,40].

Though change and alliance are mutually connected and hard to separate, analyses have been performed to try to disentangle this relation [41-43], indicating that both phenomena have separate influences on outcome. So, apart from ‘early change’, the alliance has emerged from patient-focused research as a second predictor of outcome.

There are indications that timely detection of a poor working alliance results in better outcomes and that feedback about this topic can initiate improvement/remediation of the relationship, preventing stagnation in the therapeutic process and identifying potential drop-out in good time [31,42,44].

### Results of studies on alliance on feedback

Scarce studies have been performed on the specific effect of feedback on alliance in relation to outcome (Whipple [16], Harmon [38] and Slade et al. [45], Crits Christoph [37]). Fluckiger et al. (2013) [46] found that multiple interconnected components simultaneously influence the early phase of treatment, while interpersonal distress at intake as well as the early interpersonal session experiences by patients and therapists seem to be robust predictors of outcome. These findings underscore that therapists need to monitor and discuss intra- as well as interpersonal experiences early in therapy, though its interaction is not yet fully understood.

It is yet unclear whether feedback on the alliance improves alliance, and it is equally unclear whether feedback on the alliance actually boosts outcome; let alone if these mechanisms work similar in a psychiatric and psychotherapeutic settings. Subsequently, it is unknown if applying an alliance measure to a feedback system (like in PCOMS) actually contributes to the improvement of outcome.

These considerations taken into account, it is important that a future study on feedback informed treatment adds a separate instrument for measuring the quality of the alliance in both feedback and non-feedback condition. Hence making it possible in a more independent way to examine if feedback (on alliance) actually influences the quality of the alliance; and to examine the relation between separately measured alliance scores, alliance scores provided by the feedback instrument and outcome scores.

The design of this study meets this requirement by adding the Helping Alliance Questionnaire II for both client and therapist.

In summary, we can conclude the following from the studies above.

Immediate feedback would appear to be a promising method but, at the outset of our study, studies were restricted to a few specific populations such as students and partner counselling, with the level of distress at the outset of the treatment appearing to be relatively limited (with an average ORS score of about 20). The method has not yet been studied in patients with severe and acute psychiatric symptoms.

Also, it is yet unclear if feedback on alliance improves the alliance and if this process influences outcome. In feedback studies, both outcome and alliance have not yet been examined with independent measures so far.

Taking into account all considerations mentioned before, the research questions for this study are:

1) to examine whether applying immediate feedback at each treatment session in a psychiatric population with severe distress results in better outcomes (10% fewer complaints, enhanced well-being), higher client satisfaction and a more efficient treatment (shorter treatment duration) compared with the no-feedback condition (the same therapists operating in both conditions). Outcome has to be measured with a measure independent from the instrument used in the feedback process.

2) to examine whether applying immediate feedback at each treatment session results in improvements to the alliance; and whether there is a link between any improvement in alliance and improvement in treatment outcome. Alliance to be measured with a measure independent from the instrument to be used for feedback.

## Methods

### Design

The study is a randomised controlled trial covering all patients presenting to the Crisis Intervention & Brief Therapy team over a period of two years (starting October 2009). When patients are presented to the CI&BT team, they are randomised to the experimental condition (EXP) or the control condition (TAU). The EXP condition uses an immediate feedback method for each treatment session; the TAU group does not use formalized feedback.

### Experimental intervention

The experimental condition receives treatment using the immediate continuous feedback method Partners for Change Outcome Measurement System (PCOMS).].

The therapist establishes in dialogue with the client how scores should be interpreted and how to optimize the chances of progression during the therapy sessions coming.

In the EXP condition, clients complete the ORS (‘How are you doing?’)with a member of the research staff preceding every session. The results are presented in a graph, together with the ORS scores of the previous contacts. A printed version of this graph is given to the clients, who then discuss it with their therapist. At the end of the session, the therapist asks the patient to complete the SRS ( ‘How did you experienced the meeting?’); once this has been done, the resulting feedback is discussed on the spot. The SRS is, once it has been discussed with the therapist, passed onto the researcher after the end of the session. In both conditions, patients are treated by the same therapists using the same therapeutic method and systemic crisis intervention; supplemented by the CDOI feedback method for patients in the experimental condition.

### TAU condition: treatment as usual

The treatment delivered to both the TAU and the EXP groups can be described as crisis intervention in combination with brief therapy. The approach is an integrative model in which both psychiatric treatment and systemic therapy approach play an important role. This treatment method has been described extensively in the Dutch Crisis Intervention Practice Handbook [47]. At present, there are no comparable crisis intervention treatment models that are known to have been scientifically studied [48]. The approach to treatment used is ‘practice-based’ too. The TAU for crisis cases is delivered, as in the EXP condition, by the staff of the CB&BT team of the Amsterdam mental health organisation Mentrum (part of a the Arkin institute). This team has extensive experience with helping people in crisis situations. The organisation of the team resembles that of Assertive Community Treatment teams: intensive outreach care, partial sharing of caseloads, working in co-therapy and immediate availability. Since the target group consists of patients suffering from a variety of symptoms, the therapeutic approach also covers a broad range of interventions. The content of the method applied includes elements from family-therapy, picking up on strengths and individual responsibilities in the system, and elements from the solution-driven approach. The attitude places a strong emphasis on motivational techniques.

Clients undergo standard psychiatric examination, supportive and structuring interventions and systemic interventions. Psycho-education, pharmacotherapy, a monitoring plan, change-oriented interventions or admission to a clinic are treatment options on offer.

Treatment is delivered in the community as much as possible. The basic philosophy of the CB&BT team involves supplying 'system-oriented tailored care': the optimal combination and intensity of interventions is established for each client system. These can vary during the course of the treatment. Treatment continuity is a goal; treatment is delivered as much as possible by the same two therapists. The therapists come from different disciplines: doctors (residents), psychiatrists, psychologists and psychiatric nurses. The core team consists of experienced therapists. The team is supplemented by a group of residents that changes every six months. The primary focus of the treatment is to help clients regain a sense of mastery over their lives again. However, compulsory admissions also take place when necessary if clients are unfit to make their own decisions and their behaviour is dangerous.

Therapists can ask for consultation about difficult cases any moment. A consulting psychiatrist is always available, and daily case meetings are open to any therapist who faces a problem or feels a case is not progressing. This means there is no principal difference in the quality and availability of services between TAU and Experimental Intervention.

### Randomisation

When entering the treatment facility, patients will be randomly assigned to one of both conditions. Randomisation is performed by a member of the research team before contact with the therapist takes place. There is no assessment for eligibility, all patients referred to the CI&BT team will be included in the study. Though, after the first or second assessment session, a substantial part of the clients is being referred to other services, for instance send to their GP or admitted to a hospital. Only a limited group of clients will be indicated to continue treatment at the CI&BT team. Those who continue treatment after two assessment sessions form the group that is subject to this study. At first contact it is not clear whether a client will be referred to other services or taken into treatment at the CI&BT team, while treatment has already started the moment this becomes clear. As a consequence, only after two sessions it is evident which patients will participate in the definite research condition.

So, a substantial group of randomised patients will be excluded. This post-randomisation exclusion is inevitable, given the nature of the service. Excluding patients from an analysis does not necessarily bias the results, provided that certain requirements are met, for instance that intention-to-treat applies to all randomised patients [49]. Baseline characteristics of all patients, such as gender, ethnicity, education, marital status, diagnosis and level of functioning, shall be analysed to make sure selective exclusion does not bias the results.

#### Procedures and time points

Initial assessment (the 'baseline measurement') takes place when patients first come into contact with the Crisis Intervention & Brief Therapy Team. The intervention lasts a maximum of six months and stops when treatment ends or after six months.

The PCOMS questionnaires are completed and discussed at every session in the EXP condition. In the TAU condition, every six weeks the ORS form is completed and handed over to a research assistant, without discussing it with the therapist. In both conditions, starting at the time of the baseline measurement, additional questionnaires (BSI, HAQII) are completed by patients in both conditions after 6 weeks, 12 weeks, 18 weeks and 24 weeks of treatment. The therapist also completes questionnaires at the same point in time (6, 12, 18 and 24 weeks after treatment starts) about the course of treatment (CGI) and the alliance (HAQII). When treatment ends, all questionnaires are being completed once again. The follow-up measurement takes place three months after the end of treatment.

Before the study starts, the therapists are required to complete a one-off questionnaire measuring their attitude to the instrument and their motivation with respect to the use of feedback in general. The same questionnaire will be completed by the therapists after the study period.

### Setting

The setting for this study is a Mental Health Crisis Intervention & Brief Therapy team (CI&BT team) in a large city, where patients with severe psychiatric and psychosocial problems come into contact with the CI&BT team during crisis. Therapists in this team are treating these patients on an outpatient basis for a period of a maximum of six months after the crisis.

Clients of different ages – with a minimum of 18 years – are referred by GPs, self-employed therapists, mental health outpatient clinics and the police. Treatment is given at the centre of the CI&BT team. There is no diagnostic screening beforehand: the CI&BT team starts immediately on a combination of diagnosis and treatment.

#### Participants

This is a mixed diagnostic group of patients with acute psychiatric problems: axis I conditions, problems resulting from personality disorders (axis 2 problems such as borderline or anti-social personality disorders) and serious psychosocial problems (severe family conflicts, anti-social behaviour).

The distribution of principal diagnoses in the past years (2009–2011) was as follows: 21% with a psychotic disorder, 16% with an adaptive disorder, 10% with a depressive disorder, 9% with an anxiety disorder, 8% with a disorder associated with substance abuse and 5% with a bipolar disorder, 6% with the principal diagnosis of personality disorder, 3% without a score or deferred, 1% cognitive/organic disorder, other 31%.

After ending treatment 30% of the clients entering the CI&BT team was referred to other clinics.

### Measurement instruments

#### PCOMS

PCOMS contains two very short questionnaires consisting of four items each, one for determining the outcome and the other for determining the quality of the working alliance. The patient is required to complete these questionnaires at every session. So outcome and alliance can be discussed immediately by therapist and client on a session-by-session basis. Duncan and Miller [44] developed PCOMS, two short questionnaires for obtaining feedback as simply and effectively as possible about both the alliance and the effect of the treatment. They describe the use of these two questionnaires as 'Client Directed Outcome Informed' (CDOI) practice. PCOMS is a methodical tool: therapists continue to work according to their own therapy model. Characteristically, PCOMS visually describes satisfaction with the therapeutic relationship (i.e. the alliance) and development of client well-being (i.e. the outcome), using simple scoring lists. For this purpose the ‘Outcome Rating Scale’ (ORS) and the ‘Sessions Rating Scale’ (SRS) are used. The questionnaires are completed and discussed at every session. In the slightly longer term, the ORS is an indicator of perceived effect of the treatment as a whole and, in combination with the SRS, provides a timely indication of any stagnation in the therapy. When a client has doubts about the alliance, these emerge as a low score on the SRS. A persistently poor alliance is associated with a high drop-out risk [14].

#### Outcome rating scale

The ‘Outcome Rating Scale’ (ORS), the ‘How are you?’ form, measures client assessments of various areas of their functioning. The ORS is discussed briefly at the beginning of every session. When change is evident compared to the previous session(s), the discussion fixes its eye upon the significance of that change. This discussion can be minimal or extensive depending on the information obtained. The form consists of one A4 form with four visual analogue scales all relating to an aspect of client well-being: ‘individual’, ‘relational’, ‘social’ and ‘general’. The clients are asked to place a cross or hash mark somewhere on each line as an assessment of their functioning in the period prior to the session. The far left of the line is ‘poor’; the far right is ‘good’. On a line measuring 10 cm, this results in a score between 0 and 10. Internal consistency for the English version of the ORS is .93 and validity has been demonstrated by studying the correlation with the OQ-45 [17].

#### Session rating scale

The ‘Session Rating Scale’ (SRS), the ‘What did you think of the session?’ form, measures various aspects of the alliance and it is completed at the end of the session. This also is a single form with four lines, being scored in the same way as the ORS form. It looks at four aspects of the treatment session: the relationship, the goals, the approach and the session as a whole. The questionnaires are completed and discussed at the end of every session. When the crosses on the ‘What did you think of the session?' form indicate reticence or plain dissatisfaction, questions are asked about the subject of dissatisfaction. Relatively limited criticisms can be discussed immediately. In case of structural comments or criticisms an appointment is made to discuss these at length during the following session. A check is made to see whether there is enough confidence on the client's side to ensure that the client will return for the next session.

Internal consistency of .88 has been found for the English version of the SRS, and a correlation of .48 has been found with the HAQ-II [26].

The English version of the CDOI questionnaires has now been adequately validated [50,51]. The indications for the Dutch version are that the questionnaires are psychometrically reliable [52,53]. The translation of the ORS and SRS was made by Hafkenscheid et al. [39]. Details of this translation were adapted in consultation with a number of Dutch therapists, and Duncan and Miller; a translation of the principles of the CDOI method is made by Van Oenen [54].

#### Choice of feedback instrument in this study

PCOMS elaborates on existing instruments such as the OQ 45, but offers a much shorter score list. Miller et al. [27] have argued that time-consuming procedures interfere with the implementation of feedback, therefore a short measure has an important advantage in the light of the necessity of applying session-by-session feedback.

Since there is little time and attention for scoring questionnaires in emergency psychiatry, score lists have to be short, but at the same time provide a broad spectrum of information about complaints as wel as about general wellbeing. Information about the therapeutic alliance should be gathered in an equally efficient way. Since therapy outcome and satisfaction with the alliance are measures in PCOMS using straight forward scoring lists which can be completed quickly, the ‘Outcome Rating Scale’ (ORS) and the ‘Sessions Rating Scale’ (SRS) are the preferred instrument for this setting.

This need for an easy-view instrument also applies to the therapists who have, during the crisis phase, little time and attention left for elaborate explanation and filling out forms. Also, the CI&BT team handles psychiatric as well as psychosocial problems, so therapists will prefer a feedback instrument that can refer to both aspects of a crisis situation.

### Interpretation and consequences of the ORS and SRS scores

#### ORS

When change occurs compared with the previous session, the therapist is instructed to discuss the significance of that change. Is it related to the treatment or is a life event influencing the well-being at this point? Therapists will be informed about expected treatment curves (ETC) based on empirically derived decision rules [21] and instructed to compare actual progress to the ETC. During the initial sessions (mild) improvement should be visible, the absence of any improvement is considered to be an important predictor of therapy failure and drop-out.

#### SRS

A low score on the SRS form may indicate client doubts about the alliance. This need not be a problem when the ORS form indicates that well-being is improving. Therapists are instructed to detect potential breaches in the alliance. Breaches are defined as a score less than 36 in total or less than 9 on any item of the SRS. It is made clear to the client that the SRS score intends to facilitate a conversation about the alliance between client and therapist and that no one has to be offended by the SRS scores since they offer a way for therapists to improve and tailor services based on client preferences.

When a low ORS score fails to rise after a few sessions, the case should be discussed in intervision/supervision or a colleague should be consulted. If better scores still fail to materialise after about seven sessions, referral to another therapist is the appropriate option.

Although therapists will be strongly encouraged to openly discuss the feedback with clients, the frequency or content of these interactions is not formally monitored.

Adherence to the feedback system will be stimulated by regular supervision sessions.

### Outcome questionnaire 45

The Outcome Questionnaire 45 (OQ-45) is a self-report questionnaire developed by Lambert [55] with the aim of mapping out the course of client symptoms during and after the end of the treatment by means of the structural completion of the questionnaire. The list consists of 45 statements leading to three subscales that are aimed at assessing different domains of client functioning: Symptom Distress, Interpersonal Relations and Social Role. Scores are generated for each subscale and the total score is obtained by summing up the three subscores. The internal consistency for the Total score of the Dutch OQ-45 ranges between 0.92 and 0.96 in university, community, patients and community and patients combined samples. For the subscales the consistency is 0.90- 0.95 for the Symptom Distress scale, 0.74-0.84 for the Interpersonal Relations subscale and 0.53-0.72 for the Social Role subscale [56].

### The brief symptom inventory (BSI)

The Brief Symptom Inventory [57] is the concise version of the Symptom Checklist 90 and consists of 53 statements. The BSI is a self-report questionnaire for measuring symptoms of psychopathology in adults. The patient is asked to state on a five-point scale ranging from ‘very much so’ to ‘not at all’ the extent to which the statement has been applicable to him/her during the previous week. The results lead to a general score and three sub-scales: the Global Severity Index (GSI), Positive Symptom Distress Index (PSDI), and Positive Symptom Total (PST). The questionnaire was translated from English to Dutch and then back-translated before the original version was compared with the translation. In addition, the Dutch translation was compared with the translation of the Symptom Checklist-90 and the definitive translation was then adopted after a number of changes had been made [57]. Reliability (i.e. the alpha coefficient) of seven of the eight scales was > .80 and this means that reliability is satisfactory. The only exception is the psychoticism scale (.71). The reliability of the scale as a whole is .96. Validity was examined by linking the BSI to the Symptom Checklist-90-R and a few other scales for psychopathology.

### Helping alliance questionnaire

The Helping Alliance Questionnaire (HAQ-II) is a questionnaire developed by Luborsky [58] that maps out the alliance between the patient and the therapist as perceived by the two. Both state independently, in response to 19 statements, the extent to which they agree with the description of the alliance on a six-point scale ranging from 'disagree entirely' to 'agree entirely'. Luborsky et al. report internal consistency reliabilities (alphas) of 0.90 to 0.93 and test-retest reliabilities of 0.56 to 0.78; a further study reported alphas of 0.85-0.91 [58].

The questionnaire was translated from English to Dutch and then translated blindly back to English. The Dutch version of the HAQ-II has been psychometrically evaluated [59].

### Clinical global impression

The Clinical Global Impression (CGI) [60] is a very short instrument consisting of two questions to be completed by the therapist. The CGI is mostly used for making a global assessment. The first question relates to the global severity of the patient's problems, as established on a seven-point scale ranging from ‘normal/not a problem’ to ‘very severe problem’. The second question relates to global change as compared to the status at the outset of the treatment, which can also be stated on a seven-point scale ranging from ‘major improvement’ to ‘major decline’. The CGI has been widely used in clinical research concerning social anxiety [61] and depression [62] and (most often) one and the same format of the CGI is being used for studying various sorts of pathology. The question of the validity of the CGI is still debated [61].

### Satisfaction questionnaire

This first author devised this questionnaire with the aim of establishing patient satisfaction after the termination of the treatment. The questionnaire comprises two separate components: the first is completed by all patients and consists of a five-point scale that allows patients to state the extent to which they are satisfied with different aspects of the treatment. The second component is restricted to patients in the experimental condition. It allows patients to state on a five-point scale to what extent they are satisfied about the use of PCOMS. As the questionnaire was developed especially for this research the reliability and validity haven’t been established yet.

### Attitude questionnaire

This is a questionnaire developed by Morton Anker in 2005 [31] that asks the therapists about their attitude to the PCOMS instrument (both the ORS and SRS). This is done using 19 statements and therapists are required to state the extent to which they expect the instrument to make a – positive or negative – contribution to the treatment outcome on a five-point scale ranging from ‘entirely agree’ to ‘entirely disagree’. The reliability nor the validity of this questionnaire have been researched yet.

### Ethical approval and consent

The study protocol and informed consent procedure was evaluated in 2009 by the ethics committee for Dutch Mental Health Institutions, (Kamer Noord of the METiGG) (approval nr. 9219, 1-9-2009).

The Committee concluded it to be an extremely useful and clinical relevant research project, that does not fall under the jurisdiction of the WMO (the Dutch law on scientific medical research on human beings) as the use of the applied questionnaires are common practice in mental health care. Furthermore that discussing how patients are getting on and how they experience the alliance does not add any additional burden or risk for the patient, since this in fact is a basic ingredient of every psychotherapy. As a consequence the regular clinical procedure for informed consent at the department was followed. The study was explained to the patients, written information was provided and patients were asked to participate on a voluntary basis, which was noted in the medical file.

### Data analysis

#### Sample size calculation

With two groups of 90 patients, an alpha of 0.05 (one-tailed), an effect size of about 0.3 on the Global Severity Index at 12 weeks (mean EXP group = 1.0; mean TAU = 1.3; standard deviation at week 12 is 0.80) can be detected with a statistical power of 80%. Analysis will be performed according to the intention to treat principle.

#### Primary analysis: EXP versus TAU

In the primary analysis, outcomes of the two treatment conditions will be compared using repeated measures analysis (GLM), with the number of sessions as a covariate. In this analysis, each follow-up measurement will be compared separately to the baseline measurement. Since in this design patients are nested within therapists, these nested data will also be examined in a multilevel analysis, controlling for therapist variation. The number of sessions will be included as a covariate.

#### Secondary analyses

The following secondary analyses will be presented in separate papers following the paper on the primary outcomes.

### Feedback and client and therapist variation

If some therapists are generally more effective than others, then the outcomes of clients seen by the same therapists will be correlated. Three multilevel models will be constructed to examine therapist and client effects as well as the effects of feedback on posttreatment functioning, similar to the analysis by Anker et al. [31]. In Model 1, the presence of therapist and client effects will be tested by controlling for pretreatment functioning at Level 1 and evaluating the residual intraclass correlations. To determine the variance due to therapist and client, the residual intraclass (controlling for pretreatment ratings) correlation will be calculated. In Model 2, the effect of feedback will be estimated, and in Model 3 a random slope for feedback will be added to examine if the effect of feedback varies across therapists.

### Alliance and outcome

The relationship between early change, the alliance, and outcome will be examined at posttreatment and follow-up. These relationships will be analyzed in a similar manner as described in Anker et al. [33], using multilevel analysis in which the levels of patient and therapist will be taken into account. Pretreatment scores and early change will be included as covariates. Patterns in alliance scores of clients ad therapists will be examined to determine whether different patterns of alliance development differentiate outcomes, comparing clusters using chi-square analysis (clinical significant change used as cut off score) as well as ancova analysis (outcome added as a continuous measure).

## Discussion

### Replication in other target group

Previous research has generated promising findings relating to the use of feedback in treatment. The primary distinguishing feature of this study is the target group: PCOMS has been studied previously in a student population only [29] and in couples presenting for relation therapy [30,31]. In this study, we look at a group of patients with severe psychiatric and social problems. In addition, these patients are generally going through a crisis, which is a very different situation from patients looking for psychotherapeutic assistance. An initial aim of this study is therefore to establish whether the results achieved in the past are replicated in patients with psychiatric problems in a crisis service setting. Secondly, previous studies have not used supplementary measurement instruments to assess the quality of the alliance or the level of well-being systematically. Reese et al. [29,30] use PCOMS only; Anker et al. [31] use only the Locke Wallace Marital Adjustment Test as a supplementary instrument during intake and follow-up. In this study, in addition to PCOMS, we use the BSI, OQ45, HAQ II and CGI. A second aim of this study is therefore to establish a more reliable picture of actual changes in well-being, symptom reduction and functioning, and to study the relationship between developments in the alliance, feedback and outcome measures.

### Design benefits and drawbacks

The design meets the requirements formulated by Lambert [15] and Anker et al. [31] in their study for future research into direct feedback in order to ensure that the design is as strong as possible: random allocation of patients to the different study conditions, therapists working in both study conditions, the use of different treatment methods and working with experienced (accredited) care providers.

In accordance with these principles, then, all therapists participate in both conditions to which the patients are randomised (the experimental and control conditions). They therefore treat approximately 50% of their clients using PCOMS and 50% on the basis of TAU. The benefit of this design, all therapists working in both groups, is that any differences in effectiveness between therapists cannot be a factor in differences between outcomes. Given the fact that, with regard to effectiveness, differences between therapists are larger than differences between therapeutic methods, it is important to eliminate the impact of the therapist variable in order to be able to arrive at conclusions about effects of any method [63].

Another benefit is that it is not very likely that allegiance factors (i.e. therapists being enthusiastic about the method) will affect outcomes in favour of the EXP condition because all the participants have received the same training for using PCOMS and both more, and less, highly motivated therapists deliver treatment to the EXP group. The possible drawback of this design is that, as a consequence of contaminations, any positive effects of PCOMS might not emerge as clearly. Given the fact that all therapists are working in both conditions, there is a risk that the difference between the two conditions will not be as large because the therapists in the TAU condition can ask for more feedback spontaneously, or use PCOMS less naturally in EXP condition or even forget to discuss the feedback. Another potential flaw however is that, the other way around, the design favours the EXP condition because therapists may attention more to patients in the feedback condition simply because these patients are more salient to them.

### Naturalistic setting

The fact that this study takes place in a naturalistic setting means that the data collected will be very heterogeneous in several respects. The CI&BT team does not have any threshold and clients are referred through several channels. This means that the population and the diagnostic groups are heterogeneous. The method used for Treatment As Usual is not protocolized because the problems presenting render this impossible: the nature of the problems varies widely, from psychosocial difficulties to personality disorders and psychosis. Psychiatric treatment is required – and this may require compulsory admissions or medical treatment – as well as talking therapy or social support, with contact being either individual or with the system. This means that the treatment method, duration and intensity vary considerably (in both conditions).

### Patients in crisis

Another factor is that clients are referred to the team in crisis situations. This means it is a highly vulnerable group of people in desperate circumstances who, as a result of avoidance or resistance, have passed through every safety net and who often function poorly. For those patients, providing reflective feedback focusing on the patient's own situation and the relationship with the mental health services will probably be quite challenging. Otherwise, the service works in shifts, which means that changes in staffing are sometimes inevitable and the standard approach is to work with co-therapy pairings (to safeguard both quality and continuity). So the therapeutic relationship is split up between several people, which might complicate the feedback about the alliance. Finally, the therapists are a mixed group of highly experienced permanent staff (6 psychiatrists, 10 social psychiatric nurses, two psychologists and a system therapist) and relatively experienced doctors (28 years on average, some of whom are being trained as psychiatrists) to work in the team temporarily for an average of six months; this means that specific expertise can vary considerably from therapist to therapist.

Another limitation of the study is that the feedback instrument does not provide information about the influences of social support, changes in the primary domains of a persons life and historical developments concerning the family of origin, which can be of importance for understanding the broader context of the change process [64].

## Conclusion

All these factors make it awkward to interpret findings in a consistent manner. For example, it is difficult to determine to what extent the method adopted, the number of sessions, the time elapsed or other factors such as feedback determine the treatment outcomes. It is also the question whether clients who find themselves in a crisis, often precisely because of a lack of ability to think about their own situation or to cooperate with the mental health services, can benefit from more feedback and reflection, and whether this does not actually impose an additional burden on the relationship. It is also difficult to determine the extent to which the quality of the alliance is affected by changes in the therapist and/or working in co-therapy, and whether this might have offset any possibly positive effect of feedback. The participation of a relatively large group of less experienced staff may also affect the quality of the feedback process in the sense that inexperienced staff may become unsure in crisis situations when faced by critical feedback so that the feedback has less effect or is even counter-productive. Finally, the basic treatment in the EXP and TAU groups will be delivered using the specific treatment model developed in the CI&BT team, which had traditionally put a strong emphasis on motivating, client-driven approach (e.g. approaching care avoiders), with the desires and objectives of the client and people closely concerned occupying a central position. The drawback here is that adding formal feedback through CDOI may generate less added value than in a traditional treatment setting.

## Competing interests

FJvO declares being a certified trainer in CDOI and providing workshops in applying CDOI in therapeutical settings.

## Authors’ contributions

FJvO wrote the study proposal, wrote the manuscript and lead the research project. FJvO and SS developed the study design and coordinated the data acquisition. JD and RV contributed to the development of the study design and supervised the research project. RS contributed to the development of the study design. IV coordinated the data acquisition and conducted the randomization procedure. JP developed the data-analytical strategy and conducted data-analysis. All authors provided comments on manuscript drafts and approved the final manuscript.

## Pre-publication history

The pre-publication history for this paper can be accessed here:

http://www.biomedcentral.com/1471-244X/13/331/prepub
